# Portal vein/superior mesenteric vein resection in pancreatic cancer treatment in the elderly

**DOI:** 10.1097/MD.0000000000007335

**Published:** 2017-07-07

**Authors:** Jiong-Ze Fang, Cai-De Lu, Sheng-Dong Wu, Jing Huang, Jie Zhou

**Affiliations:** a Department of Hepatobiliary Surgery, Southern Medical University, Nanfang Hospital, Guangzhou, Guangdong; b Department of Hepatopancreatobiliary Surgery, Ningbo Lihuili Eastern Hospital, Medical School of Ningbo University, Zhejiang, China.

**Keywords:** elderly, pancreatic cancer, pancreaticoduodenectomy, postoperative complication, prognosis, vascular resection and reconstruction

## Abstract

There is an increased interest in extending surgical criteria for pancreatic cancer by performing pancreaticoduodenectomy (PD) combined with portal vein (PV) or superior mesenteric vein (SMV) resection and reconstruction for borderline resectable patients. However, whether this procedure suitable for elderly patients remains unclear. Here, we studied cases of pancreatic cancer treatment in our medical center to evaluate feasibility and safety of this procedure in the elderly.

Eighty-three patients 65 years of age or older who underwent PD from January 2009 to March 2014 were divided into 2 groups: PD only (Group A, 52 cases), and PD combined with PV/SMV resection and reconstruction (Group B, 31 cases). Surgical outcomes and survival rates were compared between groups. Information regarding preoperative, intraoperative and postoperative conditions, and follow-up visits were provided. The outcomes of postoperative complications and survival rates were investigated.

No difference in the preoperative data was detected between 2 groups with the exception that the serum albumin level was significantly lower in Group B (*P* = .013), indicating more deteriorating health conditions in this group. Although intraoperative time and blood loss were higher in Group B (*P* < .001 and *P* = .048, respectively), the overall postoperative complications and survival curve showed no statistical differences between 2 groups with one exception in that there was higher incidence of intractable diarrhea in Group B (*P* = .034). The symptoms, however, resolved later on with conservative treatment. The median survival time for patients in this study was comparable to other reported PD treatments. There was zero postoperative mortality in both groups.

PD combined with PV/SMV treatment did not lead to increased morbidity and motility in elderly patients 65 years of age and above. This procedure could provide a promising opportunity for borderline resectable elderly pancreatic cancer patients.

## 1 Introduction

By 2010, the elderly population aged 65 and over had reached 118.93 million (8.92 %) in China. As citizens age, the share of elderly is predicted to rise to 12.09% by 2020.^[1]^ Age is the most significant risk factor for pancreatic cancer. Although pancreatic cancer can occur in younger patients, the incidence rises with age and peaks in the 65–75 year age group.^[2]^ Surgical resection is generally recognized as the primary treatment for early-phase pancreatic cancer that shows no distant metastasis. However, most pancreatic cancer patients are diagnosed when tumors have already progressed to adjacent regions with invasion into neighboring vascular networks. Pancreaticoduodenectomy (PD) combined with portal vein (PV) or superior mesenteric vein (SMV) resection and reconstruction (PD/PV/SMV) is the main surgical option for these patients. However, PD/PV/SMV is considered highly risky because of potential complications and uncertainties in survival benefit associated with more aggressive resection. However, some reports show that vascular resection does not adversely affect postoperative morbidity and mortality.^[3,4]^ Elderly cancer patients represent a heterogeneous group with different biological, functional, and psychosocial characteristics. There is a paucity of data whether this group of patients can survive the challenging procedure of PD/PV/SMV. As the population ages, it is likely that more elderly patients will be diagnosed with resectable or borderline resectable pancreatic cancer and require surgery. We decided to assess the feasibility and safety of PD/PV/SMV in this group of patients. We retrospectively reviewed 83 cases with patients aged 65 and over who had been diagnosed with pancreatic cancer and treated continuously at our medical center during the past 6 years. Comparing PD/PV/SMV to PD only treatment, we provide evidence indicating that PD/PV/SMV treatment for elderly pancreatic cancer patients does not increase surgical risks compared with PD alone. After surgery, these patients can have survival rates comparable to those only requiring PD.

## 2 Methods

### 2.1 Study population

The data included all cases of pathologically confirmed pancreatic ductal adenocarcinoma in patients aged ≥ 65 years who received radical resection in our medical center between January 2009 and March 2014. Eighty-three cases in total had been included in this analysis. They were then divided into 2 groups: PD only without PV/SMV resection (group A, 52 cases), and PD/PV/SMV (group B, 31 cases). These patients consisted of 55 males and 28 females in total with the median age of 70.3 years (65–83). Prior to surgery all patients underwent enhanced CT and/or MRI imagine. They had general blood tests as well as tumor marker CA199 tests. General information collected from patients included age, sex, previous medical history, albumin, and hemoglobin levels. Follow-up visits were by means of outpatient reviews and telephone consultations. The follow-ups until September 2016 were analyzed. Twelve of 83 patients had terminated their follow-up visit between the 6th and 34th months after operations, leading to the final follow-up rate of 85.5%. The median follow-up time was 47 months with a range from 3 to 65 months. This study was approved by the Ethics Committee of Ningbo Lihuili Eastern Hospital.

### 2.2 Surgical approaches

All the operations were performed by a team of surgeons specialized in hepatobiliary and pancreatic surgeries. Among the 83 patients, 70 received standard pancreaticoduodenectomy (PD) and 13 total PD (TPD). The preoperative CT/MRI imaging examinations and the actual conditions during intraoperative exploration were the determining factors for the selection of standardized PD or the extended radical resection of total PD. In PD or TPD procedures, pancreatic duodenal posterior peritoneum was the primary tissue explored to see if there was any tumor invasion or metastasis. If lymph nodes at station 16b1 were negative on frozen biopsy, standard lymphadenectomy was applied to the hepatoduodenal ligament, peripheral common hepatic artery, celiac axis (CA), and superior mesenteric arteries (SMA), together with the soft tissues on the right side. Then, en bloc resection of the pancreatic cancer was performed. On the other hand, if lymph nodes at station 16b1 were positive, extended retroperitoneal lymph node dissection was performed. In all the operations, intraoperative biopsies of the biliary duct margin, pancreas margin, and soft tissues around the common hepatic artery and on the right side of CA and SMA were performed. The duodenal margin biopsy was also performed when necessary. Under most circumstances, total PD was combined with splenectomy. In terms of alimentary tract reconstruction, a self-designed embedded pancreaticogastrostomy was the preferred practice.^[5]^

### 2.3 Vascular resection and reconstruction

Preoperative characterization of images from CT/MRI was used to diagnose whether a tumor had invaded to peripancreatic vessels, and the degree of invasion. Wedge or segmental resection of PV/SMV was performed if the tumor in the pancreatic head was inseparable from adjacent veins. In a few situations where vascular invasion was less than 1/3 of the circumference, these patients were treated with wedge-shaped excision and repair. For patients whose tumors had spread to 1/3 or more of the PV/SMV regions, end-to-end anastomosis became necessary. Under this circumstance, portions of PV and SMV invaded by tumors were fully exposed and dissected away from the surrounding visceral organs and tissues. During that time, dissected tumor tissues remained connected with the invaded PV/SMV segments. Before detaching the tumor-containing segments from the distal PV/SMV, vein clamps were applied to stop bleeding. Pathological analysis was then conducted on the vein margin.

Venous anastomosis was reconstructed with continuous 5-0 or 6-0 prolene sutures (end-to-end anastomosis). Before tying the sutures, the proximal end of the vein clamp was loosened to flush out any intravascular clots and debris or air bubbles, and to allow the lumen to regain its shape. If vein resection was shorter than 5 cm in length, primary end-to-end anastomosis was appropriate. In some cases, veins were suspended from the connective tissue at the distal ends before reconstruction to avoid tension buildup in the veins. When necessary, the hepatic falciform ligament, right peritoneal wall, and the ileocaecal fold (the root of the small-bowel mesentery) were loosened to reduce tension. If more than 5 cm of vein length was resected and the anastomosis would be under too much tension, internal jugular vein or vascular substitutes were used for grafting in the reconstruction.

When arterial resection and reconstruction became necessary, the method was the same as portal vein reconstruction using primary end-to-end anastomosis. For patients who had to undergo both arterial and venous resection and reconstruction, the arterial anastomosis was completed before the removal of the mass if possible. If vascular reconstruction could not be performed except after the removal of the mass, the venous anastomosis was performed first followed by the arterial one. Arterial anastomosis was also reconstructed with continuous 6-0 prolene sutures.

### 2.4 Definition of complications

Major complications were identified in accordance with the consensus by domestic and international experts as well as the definition from the International Study Group of Pancreatic Surgery (ISGPS).^[6–9]^ The complication of pancreatic fistula can be divided into A, B, and C 3 classes based on the definition of ISGPS. Since class A pancreatic fistula does not affect hospitalization time nor require extra attention, our analysis included classes B and C only. Biliary fistula was the case that bilirubin levels in the drain fluid exceeded that in plasma when tested 3 days after the surgery, biliary fistula may be confirmed by sonography. Chyle leak was defined as output of milky-colored fluid from a drain, drain site, or the wound on or after postoperative day 3, together with a triglyceride content ≥110 mg/dL (≥1.2 mmol/L). Gastrojejunostomy leaks were verified by gastrointestinal radiography. Intraperitoneal hemorrhage or upper gastrointestinal hemorrhage was defined as the patients who had fluctuations in blood pressure that were caused by postoperative intraperitoneal hemorrhage or upper gastrointestinal hemorrhage that required blood transfusion at 400 mL or more, or RBC transfusion of 2U or more. Intraabdominal infections were diagnosed in patients who had sustained fever beyond the 5th postoperative day with an increasing amount of white blood cells (WBC) counts and local intraabdominal lesions visible on radiological imaging. Patients with abdominal incision infection or dehiscence were because of the reason that they had pus and pockets of fluid excessive serosanguineous drainage, or wound separation. In delayed gastric emptying (DGE) complication patients, the nasogastric tube remained necessary 7 days after the surgery, or the nasogastric tube had to be reinstalled 3 days after the operation. DGE was also diagnosed via upper gastrointestinal radiography. Intractable diarrhea was diagnosed if, 1 week after the surgery, diarrhea exceeded 5 times per day for 3 days or more. Deaths that occurred during postoperative hospitalizations or within 30 days after the surgery were counted as postoperative mortality.

### 2.5 Statistical analysis

The data were presented as the mean and standard deviation or percentage of the group. The statistical analysis was conducted using SPSS 21.0. Two independent samples *t* test was used to compare the means of a normally distributed interval dependent variable for 2 independent groups. Qualitative data were tested by Pearson *χ*^2^ /Fisher exact test. Survival time was counted from the day of the surgery. The survival curves were described by Kaplan–Meier Estimate. Differences between 2 survival curves were analyzed by the log-rank test. If a *P* value was less than .05, it was considered statistically different.

## 3 Results

### 3.1 Patient preoperative conditions

As mentioned previously that pancreatic cancer patients were divided into 2 groups based on whether they underwent PV/SMV resection and reconstruction in addition to PD. Collected data (Table 1) showed that these 2 groups of patients had at the same age range with an average of 70.3 years (*P* = .758). In addition, both the groups had similar male–female ratios (*P* = .459). Preoperative examinations showed levels of serum albumin were higher in Group A compared with Group B (40.2 ± 4.1 g/L vs 37.7 ± 4.7 g/L, *P* = .013), suggesting the possibility that overall health conditions of Group B were worse than Group A. There were no differences between 2 groups in serum total bilirubin levels (*P* = .390) or jaundice (*P* = .294). Among all 83 patients, 14 had diabetes conditions, which accounted for 15.4% in Group A and 19.4% in Group B (*P* = .640). Table 1 also lists CA199 test results in patients. Although a higher proportion of patients in Group B were positive for CA199 tests (63.5% vs 77.4%, *P* = .185) with a higher mean value of CA199 levels in Group B patients (393.5 ± 628.1 vs 613.7 ± 706.0 IU/l, *P* = .144), this difference did not reach statistical significance. Percentage of patients who received neoadjuvant chemotherapy were low. In fact, only 1 in Group A and 2 in Group B received such treatment (1.9% versus 6.5% *P* = .645).

**Table 1:**
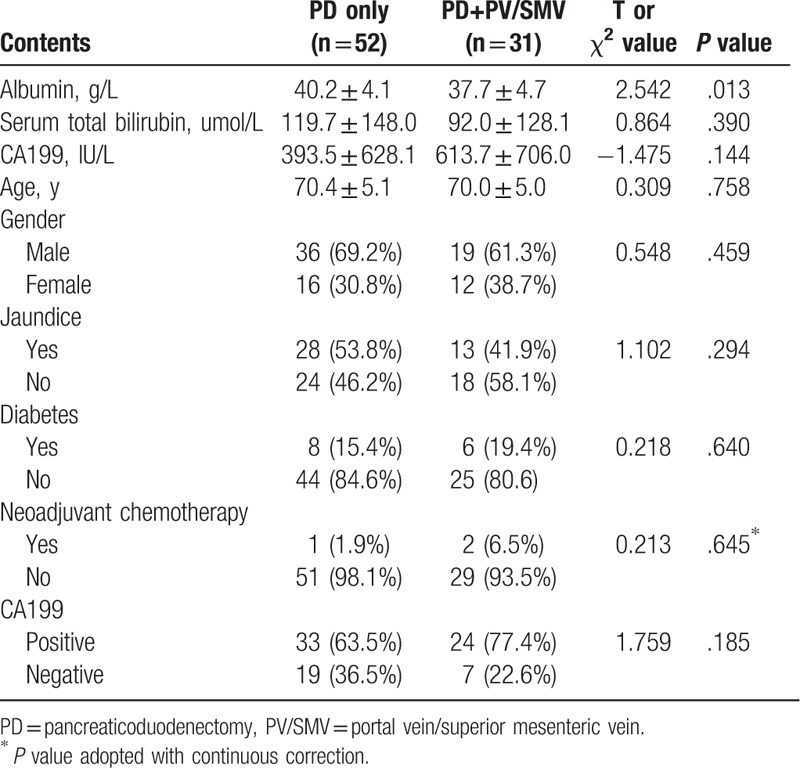
Perioperative patient characteristics.

### 3.2 Operative notes and postoperative hospitalization

As expected, patients in Group B had longer operative time (445.2 ± 92.3 min vs 369.4 ± 67.7 min in Group A; *P* < .001) (Table 2). Blood loss was also higher in Group B compared with that in Group A (945.2 ± 732.0 vs 656.7 ± 366.6 mL, *P* = .048). However, longer operations and higher blood loss did not lead to higher rates of total operative complications (*P* = .152) or mortality (which was zero in both the groups).

**Table 2:**
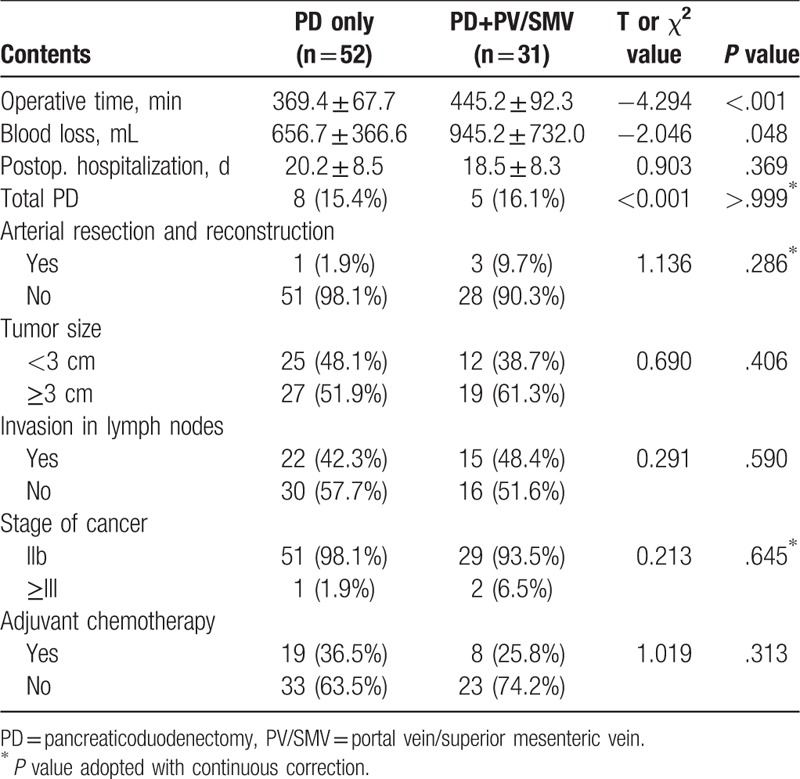
Intraoperative information, pathology data, and postoperative hospitalization.

Both the groups had a similar proportion of patients who underwent total pancreatectomy (Group A contained 15.4% and Group B 16.1%, *P* > .999). Four patients underwent arterial resection and reconstruction with 1 in Group A and 3 in Group B. In these patients, peripheral common hepatic artery and CA, but not SMA, were cut. The percentage of patients who received adjuvant chemotherapy was 36.5% in Group A and 25.8% in Group B with no statistical differences (*P* = .313). Hospitalization time after surgeries was 1.7 days longer on average in Group A but there was no statistically significant difference between the 2 groups (20.2 ± 8.5 vs 18.5 ± 8.3 days, *P* *=* .369).

### 3.3 Tumor pathology

All cases reported here are in the category of pancreatic ductal adenocarcinoma according to the UICC TNM classification of malignant tumors (7th edition).^[10]^ Pathological data of tumors in these patients showed no differences between 2 groups (Table 2). In each group, more than 50% of the patients had tumors sized at 3 cm or larger. In Group A, 48.1% of the patients had tumors smaller than 3 cm versus 38.7% in Group B (*P* = .406). Over 90% of the patients from both the groups in this study were at Stage IIb of cancer or better. One patient in group A and 2 in Group B had their cancers advanced to Stage III or worse. Furthermore, metastasis to lymph nodes occurred in 22 patients (42.3%) in Group A and 15 (48.4%) in Group B. Statistical analysis indicates that there was no difference between the 2 groups in these mentioned indices (Table 2).

### 3.4 PV/SMV resection and reconstruction

Among 31 cases in Group B, 2 had wedge resections without requiring vascular transections. The remaining patients all had PV/SMV segmental resection by removing veins varying from 1 to 5 cm in length. Among them, 10 had PD combined with splenic vein transections and ligations, including one that also underwent splenic artery ligation. All the procedures were successful without signs of postoperative splenic infarction or regional portal hypertension.

### 3.5 Postoperative complications

Zero mortality was observed perioperatively. Table 3 shows all postoperative complications. In Group A there were 11 cases, accounting for 21.2% of the group. Among these, 13.5% patients in Group A had multiple complications. The incidence of each individual complication was lower than 10% in this group. Five patients (9.6%) had to have reoperations. Although overall rate of complications was slightly higher in Group B (11 patients who accounted for 35.5%) compared with Group A, there was no statistically significant difference (*P* = .152). Similarly, if looking at individual categories of complications, the rates were slightly higher in Group B but showed no difference when compared with those in Group A (*P* > .373). One exception is that intractable diarrhea affected 4 patients in Group B, which accounted for 12.9%, while there were zero occurrences in Group A (*P* = .034). PV/SMV resection does not appear to increase reoperation rate. Three patients (9.7%) in Group B, while 5 (9.6%) in Group A, had reoperations (*P* > .999).

**Table 3:**
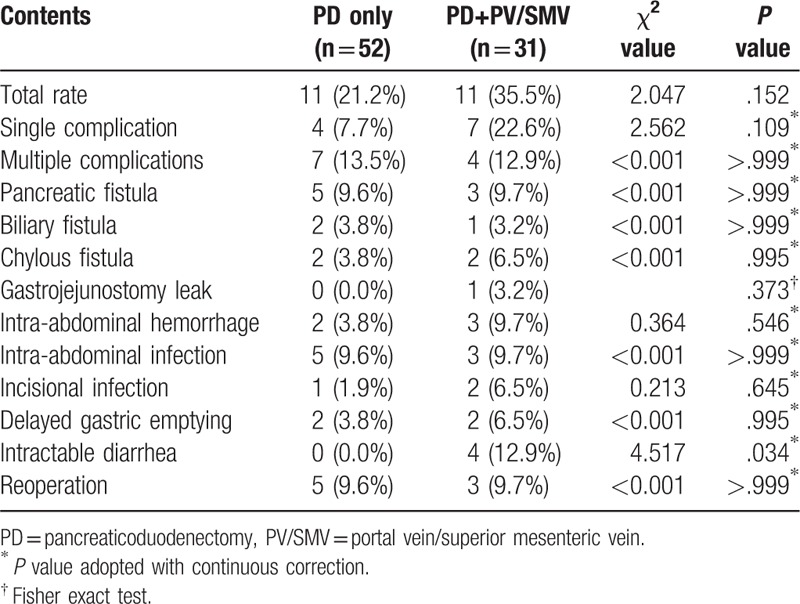
Comparison between postoperative complications.

### 3.6 Survival data

**Figure 1:**
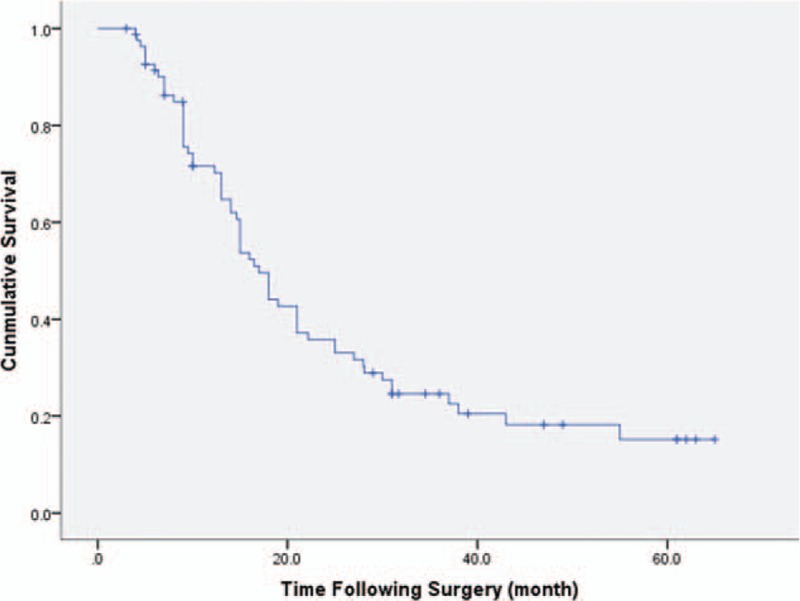
Kaplan–Meier curves of survival. All the patients in the study (Group A + Group B) were included.

Figure 1 is the Kaplan–Meier survival curve for all 83 patients in this study. The 5-year survival rate for all the 83 patients was 15.2% with a median survival time of 17 months. The 1-, 3-, and 5-year survival rates were 70.2%, 24.6%, and 15.2% respectively. Comparing Group A to Group B (Fig. 2), the median survival time in Group A was 18 months and 15 months in Group B. The 1-, 3-, and 5-year survival rates in Group A were 76.6%, 27.9%, and 23.2%, respectively; and 61.3%, 20.4%, and 10.2% in Group B. Overall, the survival curves were not statistically different between Group A and Group B (*P* = .293).

**Figure:**
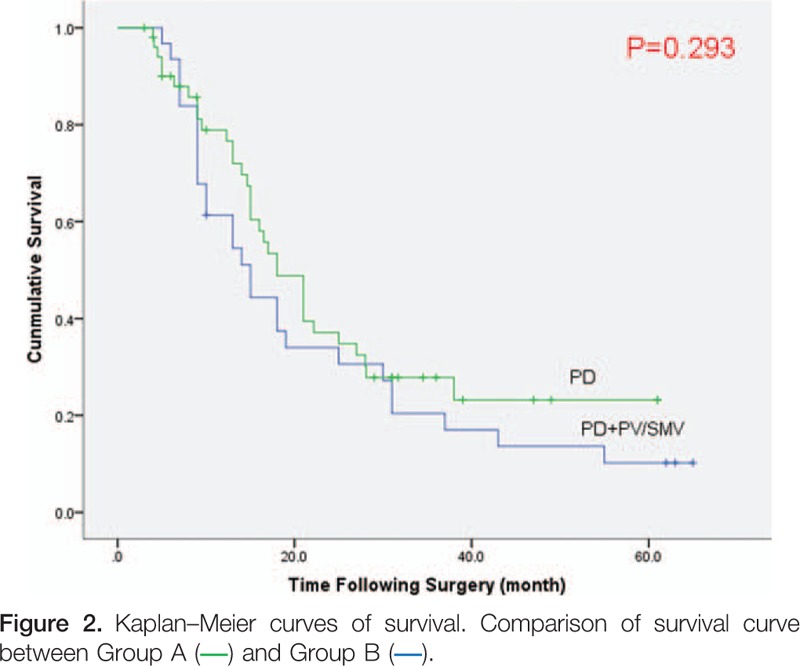


## 4 Discussion

The diagnosis of pancreatic cancer carries a very unfavorable prognosis, and surgery remains the only option for curative treatment. Unfortunately, the resectable rate of pancreatic cancer is estimated to be merely 20%.^[11]^ With improved surgical technique and experience, it is hoped that a more aggressive approach can achieve an R0 resection in more patients. PD/PV/SMV is a type of extended pancreatic cancer resection technique that dissects peripancreatic vessels and extended soft tissues to achieve R0 resection in patients whose tumor involves adjacent vascular structures. It was initiated by Fortner^[12]^ in 1973; however, it is not utilized by the majority of pancreatic surgical scholars due to its large operative trauma, high mortality rate, and unfavorable prognosis. Starting from the beginning of the 1990s, a number of reports demonstrated that, compared with standardized radical resection, extended resection of pancreatic cancer using PD/PV/SMV showed no significant increase in complication or mortality rates along with a satisfactory median survival time.^[13,14]^ However, whether PD/PV/SMV surgeries are beneficial in the elderly remains unclear. Although there are ample data indicating that PD treatments in the elderly are as safe as in younger patients,^[15–17]^ more evidence is needed to evaluate whether more aggressive PD/PV/SMV surgeries are justifiable for elderly patients.

When studying elderly patients, different subpopulations may have varied outcomes. For instance, patients in their 80s have been shown to have increased morbidity and mortality when undergoing PD surgeries.^[18,19]^ Others found that for patients in their 70s, the morbidity and mortality of PD surgeries (PD/PV/MV was included in the PD group) was similar to that of younger patients. The consensus is that age should not be a limiting factor but overall health condition may be. However, this concept urgently needs supportive data. Our analysis provides direct evidence for the feasibility of PD/PV/SMV in elderly patients. Our data indicate that properly selecting elderly patients who are physically fit for peripancreatic vascular resection can achieve acceptable postoperative morbidity and mortality rates.

The results presented in this study have indicated that it is true that the procedure of PD/PV/SMV takes more surgical time and blood loss. However, the procedure can be performed smoothly in elderly without mortality during the perioperative period. In our medical center, we did not observe increased complications in Group B with the exception of intractable diarrhea. One possible explanation is that PV/SMV procedures have a higher likelihood of removing or disturbing sympathetic nerves than PD only procedures, leading to an increased risk for intractable diarrhea. Fortunately, the symptoms resolved with conservative treatment.

An important finding from our analysis is that although the overall health conditions in Group B were not as good as those in Group A as reflected by lower serum albumin levels in Group B patients, PD/PV/SMV does not lead to an increase in operative mortality nor surgical complications. The rates of single and multiple complications were similar between 2 groups of patients (*P* = .109 and *P* > .999, respectively). In addition, the overall survival curves between Group A and Group B showed no statistical difference. This finding is remarkable because it shows that patients with more extensive tumor invasion and presumably lower health condition can achieve similar outcome to patients without vascular invasion.

In this study, we did not observe vascular complications including stenosis and thrombosis directly associated with resection and reconstruction of PV/SMV, even though increased surgical time and blood loss of PD/PV/SMV increase the risk. Surveillance was performed via Doppler ultrasound examinations of peripancreatic vessels a week after surgery, as well as with enhanced computed tomography scan 6 months postoperatively, with no evidence of these complications. However, it is possible that patients developed vascular complications after 6 months.

The goal of PD/PV/SMV is to improve the prognosis of patients. On the basis of postoperative pathology, Nakagohri et al^[20]^ divided patients with PD/PV/SMV into 2 subgroups: patients with tumor invasion into the vascular tunicae and patients with tumors distributed to the surface of blood vessels. The follow-up visit results showed that the prognosis of the former was notably worse than the latter. In clinical practice, patients with PV tumor thrombus or PV full-layer infiltration were usually diagnosed with hepatic metastasis soon after the surgery. However, before resecting the peripancreatic vessels, it is difficult to determine if or which vascular tunica was invaded by tumors. Cases such as infiltration in perivascular tissues or invasion to tunica adventitia, or merely inflammatory accretion, were resectable. It is clear that preoperative diagnosis has to be strengthened to improve prognosis. This requires us to enhance evaluation of tumor infiltration based on CT/MRI data, increase precision on pathological analysis of tumor invasion, and achieve a better identification of positive margins. By doing so, we can have improvement of prognosis by incorporating neoadjuvant chemotherapy treatments in certain patients prior to aggressive resection.^[21]^

All the data and statistics presented here have shown that regardless of the fact that PD/PV/PMV surgery may be more challenging for the patient due to longer operation time and more blood loss, it can potentially improve the prognosis for elderly patients with cancers that were previously deemed unresectable. However, surgical techniques and intraoperative management play important roles in procedure success. For example, it has been reported that even with 6 to 10 cm length of vascular transection, direct anastomosis was still applicable.^[22]^ However, experience from our practice is that primary end-to-end anastomosis is mainly successful when the vascular defects were less than 5 cm in length. When the defects were longer than 5 cm, dissociation of hepatic and mesentery ligaments is needed to avoid overstretching vessels. Sometimes, we even dissociated the splenic vein to increase the freedom of 2 vascular ends to achieve a tension-free anastomosis. Other approaches include using allograft or autologous vessel grafts for the anastomosis. In this study, 10 patients had splenic vessels divided. None of them had appearance of splenic infarction or regional portal hypertension. Therefore, splenic vein division can be considered safe although further verification with a larger amount of evidence is needed.

For all 83 cases under this study, the total complication rate was 26.5% and the operative mortality was zero. It is our view that extended resections such as PD/PV/SMV can be adopted for pancreatic cancer treatment if invasion is limited in PV/SMV regions, but physical conditions and nutritional status of the patient, especially for the elderly, should be fully evaluated so that the patient is able to physically sustain threats of complications after the surgery. Our data suggest that PD/PV/SMV resection and reconstruction is generally a safe and feasible approach even for the elderly who are physically fit. With PD/PV/SMV, it is possible to improve resectable rates and survival time of patients while strict patient selection criteria should be followed to weigh surgical risks with long-term survival benefits.
